# Dynamic comparison of early immune reactions and immune cell reconstitution after umbilical cord blood transplantation and peripheral blood stem cell transplantation

**DOI:** 10.3389/fimmu.2023.1084901

**Published:** 2023-04-11

**Authors:** Xuxu Zhao, Wenya Wang, Shiqin Nie, Liangquan Geng, Kaidi Song, Xinyi Zhang, Wen Yao, Ping Qiang, Guangyu Sun, Dongyao Wang, Huilan Liu

**Affiliations:** ^1^ Department of Hematology, the First Affiliated Hospital of USTC, Division of Life Sciences and Medicine, University of Science and Technology of China, Hefei, Anhui, China; ^2^ Blood and Cell Therapy Institute, Division of Life Sciences and Medicine, University of Science and Technology of China, Hefei, Anhui, China; ^3^ Anhui Province Key Laboratory of Immunology in Chronic Diseases, Bengbu Medical College, Bengbu, Anhui, China; ^4^ Department of Transfusion, the First Affiliated Hospital of USTC, Division of Life Sciences and Medicine, University of Science and Technology of China, Hefei, Anhui, China

**Keywords:** UCBT, PBSCT, GvHD, immune cell reconstitution, immune reactions

## Abstract

Umbilical cord blood transplantation (UCBT) and peripheral blood stem cell transplantation (PBSCT) are effective allogeneic treatments for patients with malignant and non-malignant refractory hematological diseases. However, the differences in the immune cell reconstitution and the immune reactions during initial stages post-transplantation are not well established between UCBT and PBSCT. Therefore, in this study, we analyzed the differences in the immune reactions during the early stages (days 7-100 post-transplantation) such as pre-engraftment syndrome (PES), engraftment syndrome (ES), and acute graft-versus-host disease (aGVHD) and the immune cell reconstitution between the UCBT and the PBSCT group of patients. We enrolled a cohort of patients that underwent UCBT or PBSCT and healthy controls (n=25 each) and evaluated their peripheral blood mononuclear cell (PBMC) samples and plasma cytokine (IL-10 and GM-CSF) levels using flow cytometry and ELISA, respectively. Our results showed that the incidences of early immune reactions such as PES, ES, and aGVHD were significantly higher in the UCBT group compared to the PBSCT group. Furthermore, in comparison with the PBSCT group, the UCBT group showed higher proportion and numbers of naïve CD4^+^ T cells, lower proportion and numbers of Tregs, higher proportion of CD8^+^ T cells with increased activity, and higher proportion of mature CD56^dim^ CD16^+^ NK cells during the early stages post-transplantation. Moreover, the plasma levels of GM-CSF were significantly higher in the UCBT group compared to the PBSCT group in the third week after transplantation. Overall, our findings demonstrated significant differences in the post-transplantation immune cell reconstitution between the UCBT and the PBSCT group of patients. These characteristics were associated with significant differences between the UCBT and the PBSCT groups regarding the incidences of immune reactions during the early stages post transplantation.

## Introduction

Hematopoietic stem cell transplantation (HSCT) is an effective treatment for patients with malignant and non-malignant refractory hematologic diseases (1). HLA-matched related donors are the gold standard for transplantation (2). However, only 30% of patients requiring HSCT can find HLA-matched related donors (3, 4). Umbilical cord blood is a potential source of hematopoietic stem cells (HSCs) for allogeneic transplantation and is associated with relevant biological and immunological characteristics such as low immunogenicity and highly proliferative nature of the constituent HSCs and hematopoietic progenitor cells (5–8). Furthermore, umbilical cord blood collection is easy and harmless to the donor. Moreover, it can be obtained rapidly. Therefore, UCBT is extensively used worldwide. However, because of the low stringency of HLA matching, patients that undergo UCBT may demonstrate strong graft-versus-leukemia (GVL) effects and higher incidences of aGVHD (9, 10).

Immune reconstitution after HSCT is dominated by homeostatic peripheral expansion of the graft-derived cells during the initial stages post-transplantation (11, 12). Differences in the immune reconstitution are expected between UCBT and PBSCT because they represent two diverse sources of HSCs and progenitor cells. Therefore, the success of the allo-HSCT treatment is dependent on the incidence of GVL effects exerted by the donor CD4^+^ T cells, CD8^+^ T cells, and NK cells during the immune cell reconstitution process post-transplantation (13–15). PES, ES, and aGVHD are the most common immune responses during the early stages post-HSCT (16). PES and ES are often presented as non-infection or non-drug-related fever, rashes, diarrhea, and other clinical symptoms such as pulmonary infiltrates, elevated bilirubin levels, and/or weight gain. PES occurs before neutrophil engraftment whereas ES occurs during neutrophil engraftment. However, the underlying pathophysiological mechanisms of PES and ES is poorly understood. Several reports suggest that PES and ES are caused by cytokine storm during the post-transplantation immune cell reconstitution process (17–20). However, concrete evidence is lacking from immunological studies regarding the underlying mechanisms that cause these adverse effects. Furthermore, in many cases, aGVHD cannot be distinguished from PES or ES because of overlapping pathology and mechanisms (16, 18, 20–22).

In this study, we compared the immune reactions and the immune cell reconstitution characteristics during the early stages post-transplantation between the UCBT and the related PBSCT group of patients. We also used flow cytometry to analyze the dynamic changes in the functional status and the subpopulation frequency of different types of immune cells in the peripheral blood of patients after UCBT and PBSCT. Furthermore, we used ELISA to analyze the plasma levels of cytokines (IL-10 and GM-CSF) in patients that underwent UCBT and PBSCT.

## Materials and methods

### Patients and sample collection

In this study, we enrolled 50 patients who underwent HSCT at the First Affiliated Hospital of the University of Science and Technology of China (Hefei, Anhui, China) between July 2021 and February 2022. The baseline characteristics of the patients are shown in **Table 1**. All patients were consecutively enrolled *a priori* and monitored with prospective blood sample collection and received a myeloablative conditioning regimen without anti-human thymocyte globulin (ATG). For patients receiving HLA-mismatched donors in the PBSCT group, the conditioning regimen included fludarabine (30 mg/m^2^ daily on days −8 to −5), intravenous busulfan (3.2 mg/kg daily on days −7 to −4), cyclophosphamide (CY, 14.5 mg/kg daily on days −3 to −2), and total body irradiation (4 Gy on day −1). GVHD prophylaxis consisted of post‐transplant cyclophosphamide (PT‐CY, 50 mg/kg daily on days +3 and +4), cyclosporine, and mycophenolate mofetil. The conditioning regimen for all other patients in the UCBT and PBSCT groups included fludarabine (30 mg/m^2^ daily on days −8 to −5), intravenous busulfan (3.2 mg/kg daily on days −7 to −4), cyclophosphamide (CY, 60 mg/kg daily on days −3 to −2); GVHD prophylaxis included cyclosporine and mycophenolate mofetil. All patients in the UCBT group received single cord blood transplantation. Peripheral blood samples were obtained on days 7, 10, 14, 21, 28, 35, 42, 60, and 100 after allogeneic HSCT (all-HSCT). The peripheral blood samples from healthy subjects (n=25) were used as normal controls. All the patients or their guardians provided written informed consent. This study was approved by the Ethics Committee of the University of Science and Technology of China. The clinical trial registration number for this study was ChiCTR2000041536.

**Table 1:** Baseline characteristics of the study patients.

Parameters	UCBT (N=25)	PBSCT (N=25)	*P*
Age, median (range), y	18 (0-62)	29 (4-55)	
Sex, n (%)			0.758
Males	17 (56)	18 (72)	
Females	8 (32)	7 (28)	
Disease, n (%)			0.042
AML	18 (72)	9 (36)	
ALL	3 (12)	5 (20)	
MDS	3 (12)	4 (16)	
Others	1 (4)	7 (28)	
TBI in conditioning, n (%)			0.054
Yes	1 (4%)	7 (28%)	
No	24 (96%)	18 (72%)	
Matched HLA loci, n (%)			0.249
5/10	0	7 (28)	
6/10	2 (8)	2 (8)	
7/10	7 (28)	2 (8)	
8/10	7 (28)	0	
9/10	7 (28)	2 (8)	
10/10	2 (8)	12 (48)	
GVHD prophylaxis, n (%)			< 0.001
CsA + MMF (HLA-matched donors)	2 (8)	12 (48)	
CsA + MMF (HLA-mismatched donors)	23 (92)	0	
CsA + MMF + PTCY (HLA-matched donors)	0	0	
CsA + MMF + PTCY (HLA-mismatched donors)	0	13 (52)	
Recipient CMV serostatus pre-HSCT, n (%)			0.636
Positive	22 (88)	23 (92)	
Negative	3 (12)	2 (8)	
Median prefrozen TNC, ×107/kg (range)	3.40 (1.81-13.68)	123.50 (87.80-261.60)	< 0.001
Median prefrozen CD34+ cells, ×105 /kg (range)	2.49 (1.38-6.04)	66.40 (38.20-133.90)	< 0.001
Median prefrozen CD3+ cells, ×106 /kg (range)	4.95 (0.67-30.7)	190.50 (30.50-571)	< 0.001
Median prefrozen CD56+ cells, ×106 /kg (range)	2.45 (0.49-10.12)	82.25 (7.50-362.30)	< 0.001

AML, acute myeloid leukemia; ALL, acute lymphoblastic leukemia; MDS, myelodysplastic syndrome; TBI, total body irradiation; HLA, human leukocyte antigen; GVHD, graft-versus-host disease; CsA, cyclosporin A; MMF, mycophenolate mofetil; PTCY, post‐transplant cyclophosphamide; CMV, Cytomegalovirus; HSCT, hematopoietic stem cell transplantation; TNC, total nucleated cell.

### Definition of early immune reactions

The early immune reactions after HSCT were classified in this study as PES, ES, and aGVHD during the 100 days after HSCT as reported previously (16, 23).

### Plasma sample preparation

Blood specimens were collected by venous puncture in a 5ml tube containing heparin. They were centrifuged at 400 g for 5 min at 4°C and the plasma samples were collected into sterile tubes and stored at -80°C.

### ELISA assay

ELISA kits were used to estimate the plasma levels of IL-10 (Cat. No.1111002; Dakewe Biotech Co., Ltd., Shenzhen, China) and GM-CSF (Cat. No. 1117302; Dakewe Biotech Co., Ltd., Shenzhen, China) according to the manufacturer’s instructions. The absorbances were measured at 450 nm in a microplate reader (ELX800, BioTek, Vermont, USA).

### Flow cytometry

The peripheral blood mononuclear cells (PBMCs) were isolated from the fresh blood samples and stained with fluorochrome-conjugated antibodies against several cell surface markers. After staining, the cells were washed and resuspended in PBS. In case of FACS analysis for intracellular molecules, after labeling the cell surface molecules, the cells were fixed and permeabilized with the eBioscience™ Foxp3/Transcription Factor Staining Buffer Set (Cat. No. 00-5523-00, Invitrogen, Carlsbad, California, USA), and then incubated with the fluorochrome-conjugated antibodies against the intracellular molecules (24, 25). Flow cytometry was performed using the BD LSRFortessa Flow Cytometer (BD Biosciences, San Jose, CA, USA) and the data was analyzed with the FlowJo 10 software (FlowJo LLC, Ashland, OR, USA). All the antibodies used in the FACS analyses are shown in **Supplementary Table 1**. The cell gating strategies used in the FACS analyses are shown in **Supplementary Figure 1**.

### Statistical analysis

For early immune reactions, relapse, NRM, and primary graft failure were treated as competing risks. For NRM, relapse was treated as a competing risk; and for relapse, NRM was also treated as a competing risk. The cumulative incidences of these clinical outcomes were evaluated with the use of cumulative incidence curves to accommodate competing risks and were tested using the Grey’s test (26–29). The statistical analysis was performed with the SPSS 26.0 software (IBM SPSS Statistics for Windows, Armonk, NY, USA) and the GraphPad Prism 8 software (GraphPad Prism software, San Diego, CA, USA). The statistical significance of the differences between two groups were determined using the unpaired Student’s t test. Mann–Whitney U test was used to determine statistical differences between groups for the continuous variables. The data were represented as means ± SEM. The significant statistical differences are denoted as *p < 0.05, **p < 0.01, and ***p < 0.001. Comparison of immune reconstructions using unpaired t-tests or non-parametric tests yielded almost identical results with the same conclusions. Details of the data using non-parametric tests and presented as medians with range can be viewed in the **Supplementary Table 2**.

## Results

### Early immune reactions are significantly higher in patients that underwent UCBT

**Figure 1:**
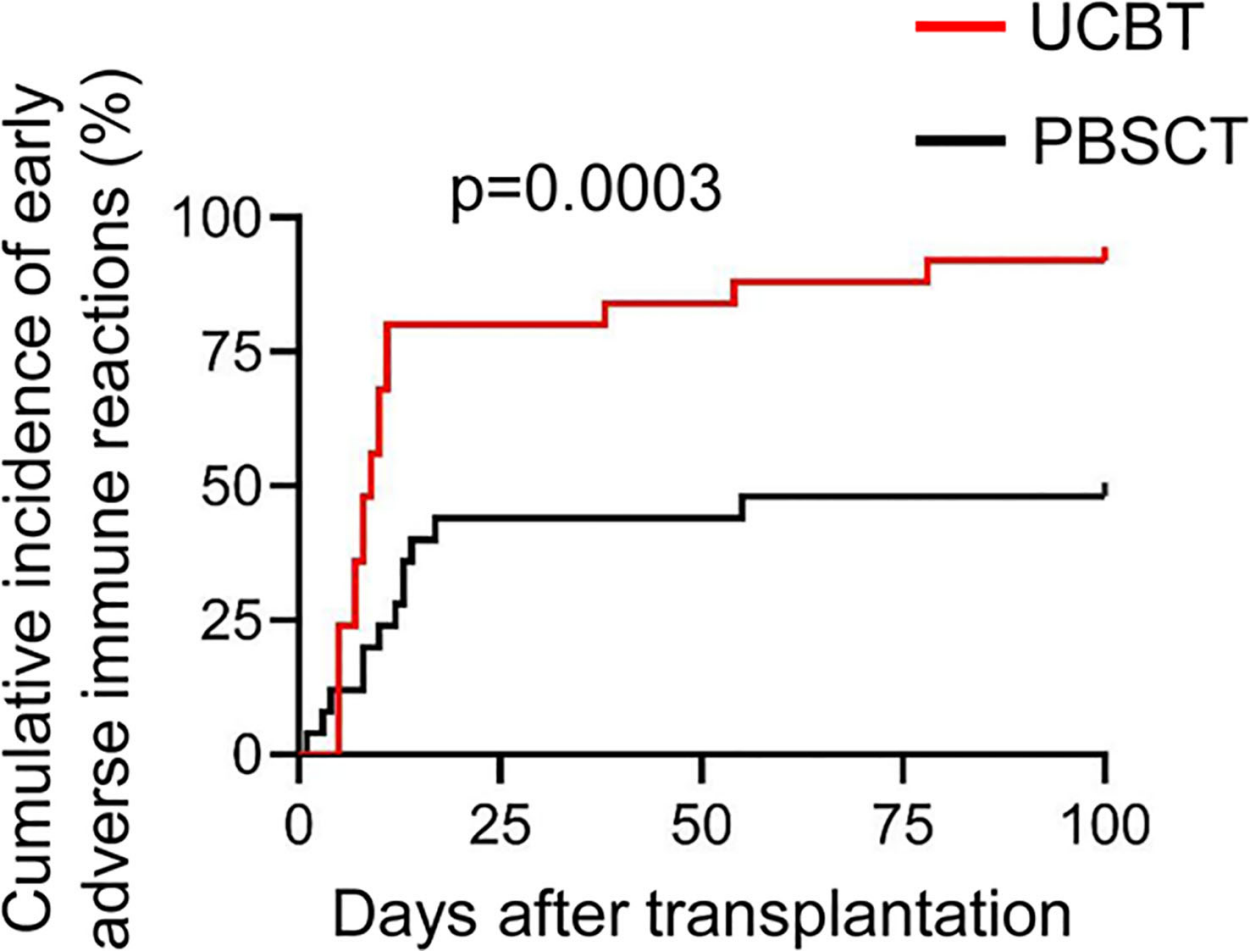
The cumulative incidences of adverse immune reactions during the initial stages after transplantation in patients that underwent UCBT or PBSCT.

The incidences of early immune reactions between the two groups of patients were assessed based on the diagnostic criteria for ES and the grading criteria for aGVHD as reported previously (30, 31). In our cohort of patients, the cumulative incidences of early immune reactions such as PES, ES, and aGVHD were significantly higher in the UCBT group compared to those in the PBSCT group (**Figure 1**). In the cohort of patients who underwent PBSCT (n=25), 8 (32%) patients developed PES and/or ES and 10 (40%) patients developed aGVHD. In the cohort of patients that underwent UCBT, 20 (80%) patients developed PES and/or ES and 15 (60%) patients developed aGVHD.

### UCBT patients showed higher frequencies and numbers of naïve CD4^+^ T cells

The rate of immune reconstitution after HSCT including the quantity and the distribution of various immune cell subpopulations influences the clinical outcomes. Therefore, we compared the dynamics of the early immune cell reconstitution process in patients that underwent UCBT and PBSCT.

**Figure 2:**
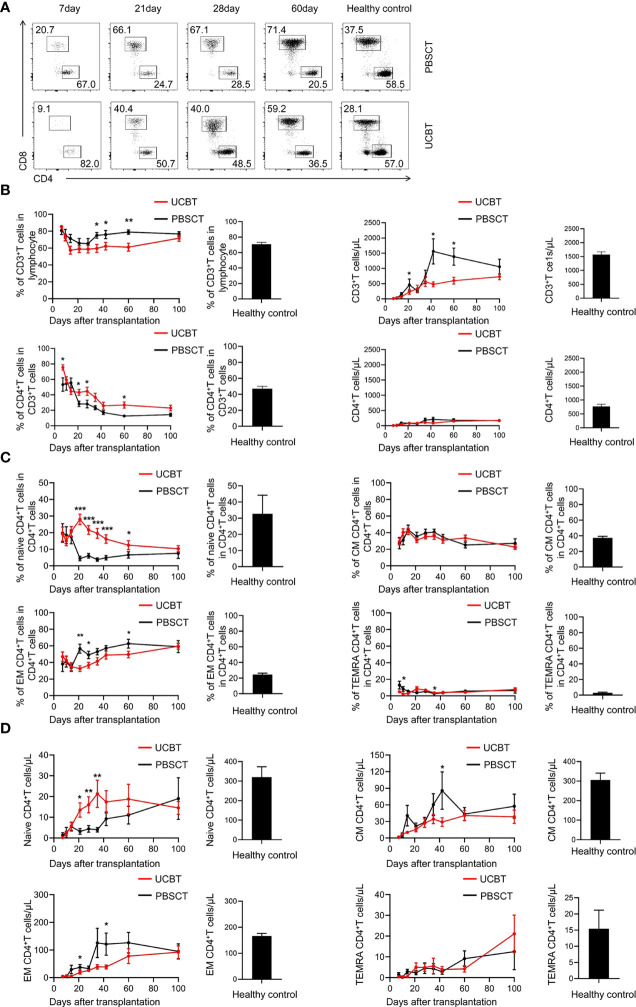
The expansion of CD4^+^ T cell subpopulations during the initial stages after UCBT or PBSCT. **(A-D)** Flow cytometry analysis of the peripheral blood mononuclear cells (PBMCs) in the UCBT and PBSCT group on days 7, 10, 14, 21, 28, 35, 42, 60, and 100 after HSCT. **(A)** Representative flow cytometry dot plots and the frequencies (percentages) of CD4^+^ T cells and CD8^+^ T cells during the early posttransplant period (day 7 to day 100) after UCBT and PBSCT. **(B)** The frequencies and the numbers of CD3^+^ T cells and CD4^+^ T cells during the early posttransplant period after UCBT and PBSCT. **(C)** The frequencies and **(D)** numbers of naïve CD4^+^ T cells, central memory CD4^+^ T cells, effector memory CD4^+^ T cells and terminally differentiated effector memory CD4^+^ T cells during the early posttransplant period after UCBT and PBSCT. The histogram shows data for the healthy control group. CM, central memory; EM, effector memory; TEMRA, terminally differentiated effector memory. The data was analyzed by the Mann–Whitney U test and represented as means ± standard error of the mean (SEM). *p < 0.05; **p < 0.01; ***p < 0.001.

T cells play a central role in the pathogenesis of GVL and GVHD. Therefore, we first analyzed the differences in the reconstitution of the peripheral blood T cells between the UCBT and PBCT groups (**Figure 2A**). The PBSCT group patients showed a faster rate of T cell reconstitution than the UCBT group, with a higher frequency and numbers of CD3^+^ T cells, which reached normal levels in the second month after PBSCT (**Figure 2B**). Furthermore, the number of CD4+ T cells showed slower recovery in both two groups compared to the CD8+ T cells (**Figures 2B**, **5A**).

The frequency of CD4+ T cells among CD3+ T cells decreased gradually from about 80% at 7 days after transplantation to a low level of 20% at 100 days (**Figure 2B**). Then, the numbers of CD4+ T cells gradually increased to 200 cells/ul, but remained significantly lower than the normal level until the 100th day after HSCT. However, the UCBT group had a higher proportion of CD4+ T cells in CD3+ T cells than the PBSCT group within 100 days (**Figure 2B**).

We then assessed the reconstruction of the CD4+ T cell subpopulations. T cell differentiation is characterized by the expression of surface markers, CD45RA and CD62L, which distinguish between naïve T cells, central memory T cells, effector memory T cells, and terminal differentiated memory T cells (32). CD4+ T cell expansion is characterized by memory T-cell skewing (33). In both two groups, we observed expansion of the effector memory CD4+ T cells and gradual reduction of the frequency of naïve CD4+ T cells gradually decreased within 100 days (**Figure 2C**). The UCBT group showed a higher frequency and numbers of naïve CD4+ T cells and a lower frequency and numbers of effector memory CD4+ T cells during the early stages post-transplantation compared to the PBSCT group (**Figures 2C, D**). This suggested that the higher frequency of CD4+ T cells in the UCBT group patients was due to a higher frequency of the naïve CD4^+^ T cells. Taken together, these data suggested that the frequency and numbers of naïve CD4^+^ T cells were significantly higher during the initial stages post-transplantation in the UCBT group compared to the PBSCT group.

### The UCBT group showed significantly lower frequency and numbers of CD25^+^CD4^+^FOXP3^+^ Tregs

**Figure 3:**
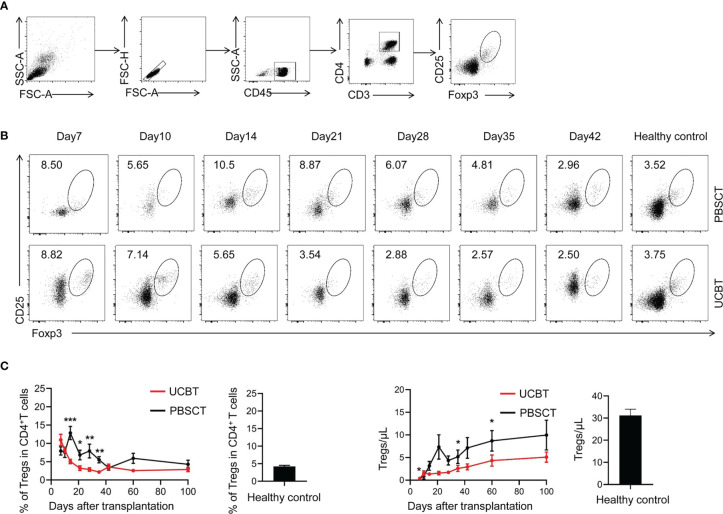
The UCBT group show a lower percentage and numbers of CD25^+^CD4^+^FOXP3^+^ Tregs compared to the PBSCT group. **(A)** Representative flow cytometry dot plots and the frequencies of CD25^+^CD4^+^FOXP3^+^ Tregs on days 7, 10, 14, 21, 28, 35, and 42 after UCBT and PBSCT. **(B)** The frequencies and the numbers of CD25^+^CD4^+^FOXP3^+^ Tregs within 100 days after UCBT and PBSCT. The data was analyzed using the Mann–Whitney U test and represented as means ± SEM. *p < 0.05; **p < 0.01; ***p < 0.001.

The CD4+CD25+FOXP3+ cells represent a subpopulation of CD4+ T cells called as T regulatory cells (Tregs), which suppress immune responses (34). We analyzed the dynamics of the immunosuppressive Tregs after HSCT in the UCBT and PBSCT groups (**Figures 3A, B**). Our data demonstrated that the frequency and numbers of CD25+CD4+FOXP3+ Tregs were significantly lower in the UCBT group compared to the PBSCT group (**Figure 3C**). Until 100 days after transplantation, the frequency of the CD25+CD4+FOXP3+ Tregs of the two groups continued to decrease to nearly 5%. However, the frequency of Tregs was significantly lower in the UCBT group compared with the PBSCT group, especially in the first 42 days. Then, the number of Tregs gradually increased until the 100th day after HSCT, but the numbers were still significantly lower compared to the healthy subjects. Furthermore, the numbers of Tregs were significantly lower in the UCBT group compared to the PBSCT group at all time points, especially during the initial stages after transplantation.

### UCBT group showed increased proportion of highly active CD8^+^ T cells

**Figure 4:**
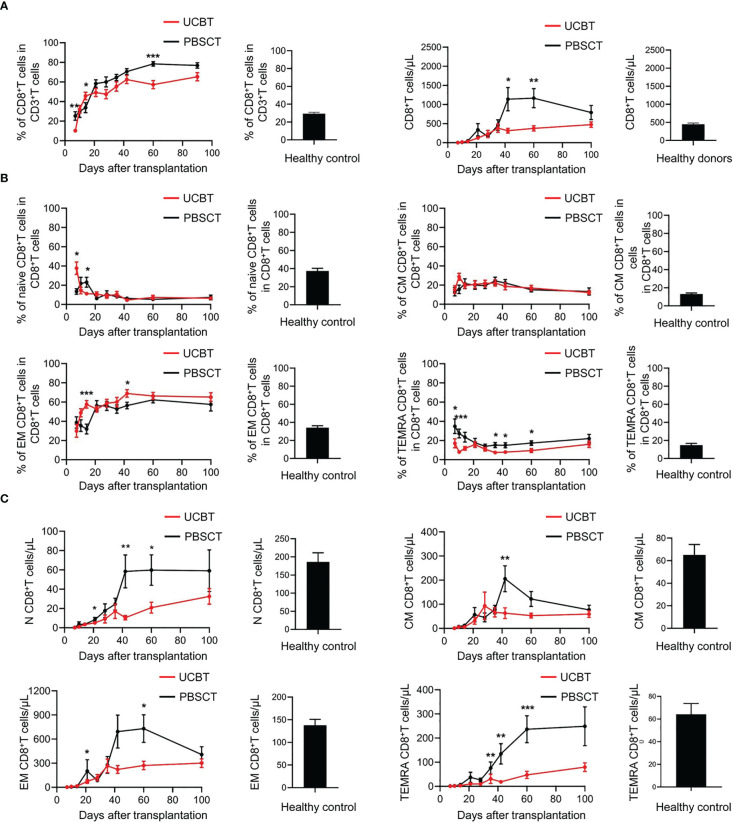
The dynamic changes of CD8^+^ T cell subpopulations in UCBT and PBSCT group patients. **(A)** The frequencies of CD8^+^ T cells during the early posttransplant period after UCBT and PBSCT. **(B)** The frequencies and **(C)** the numbers of CD8^+^ T cells subpopulations during the early posttransplant period after UCBT and PBSCT. The data was analyzed by the Mann-Whitney U test and represented as means ± standard error of the mean (SEM). *p < 0.05; **p < 0.01; ***p < 0.001.

Next, we analyzed the dynamics of the CD8+ T cells after HSCT in the two groups of patients. The frequency and numbers of CD8+ T cells significantly increased to higher than normal levels within 42 days (**Figure 4A**). Moreover, the increased frequency of CD8+ T cells was more pronounced in the PBSCT group compared to the UCBT group. We then analyzed its four subpopulations of CD8+ T cells, namely, naïve (CD45RA+CD62L+CD8+ T), central memory (CD45RA-CD62L+CD8+ T), effector memory (CD45RA-CD62L-CD8+ T), and terminal differentiated memory CD8+ T cells (CD45RA+CD62L-CD8+ T) (**Figure 4B**). We observed significant expansion of the effector memory CD8+ T cells among the CD8+ T cell subpopulations after HSCT. The PBSCT group showed significantly higher frequency of TEMRA CD8+ T cells compared to the UCBT group. Furthermore, the recovery of the four subpopulations of CD8+ T cells, especially TEMRA CD8+ T cells, was faster in the PBSCT group than in the UCBT group (**Figure 4C**).

**Figure 5:**
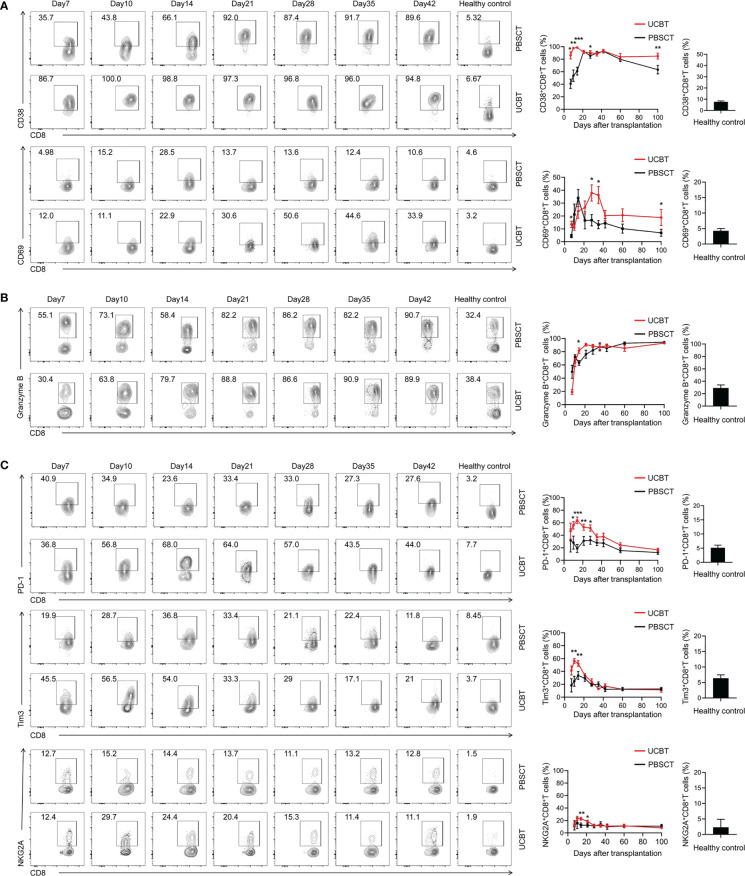
The dynamic changes of functional molecules expression levels in the CD8^+^ T cell subpopulations with UCBT and PBSCT. Representative flow cytometry contour plots and the dynamic changes in the expression levels of **(A)** CD38, CD69, **(B)** GZMB, **(C)** PD-1, Tim3 and NKG2A in the gated CD8^+^ T cells during the early posttransplant period after UCBT and PBSCT. The histogram shows data for the healthy control group. CM, central memory; EM, effector memory; TEMRA, terminally differentiated effector memory. The data were analyzed by the Mann–Whitney U test and represented as means ± SEM. *p < 0.05; **p < 0.01; ***p < 0.001.

Immune responses depend not only on the numbers of specific immune cells, but also on the qualitative features of the immune cells. Therefore, we analyzed the expression levels of functional molecules in the CD8+ T cell populations. The expression levels of immune-activating surface molecules such as CD38 and CD69 on the CD8+ T cells were significantly increased after transplantation compared to the healthy subjects (**Figure 5A**). Furthermore, in the UCBT group, the expression levels of CD38 on the CD8+ T cells were higher in the first three weeks and the expression levels of CD69 on the CD8+ T cells were higher in the fourth and fifth weeks after transplantation compared to the PBSCT group. The intracellular expression levels of granzyme B, a molecule critical for the cytolytic function of the CD8+ T cells, was significantly elevated in the patients that underwent HSCT compared to the healthy subjects (**Figure 5B**). Moreover, the expression levels of immunosuppressive molecules such as PD-1, Tim-3, and NKG2A were significantly higher in the patients after HSCT, especially those that underwent UCBT compared to the healthy subjects (**Figure 5C**). However, the expression of these immunosuppressive molecules gradually decreased over time until 100 days after transplantation. Collectively, these data showed that the activation of CD8+ T cells was significantly higher in the UCBT group compared to the PBSCT group.

### UCBT group showed significantly increased numbers of highly activated NK cells

**Figure 6:**
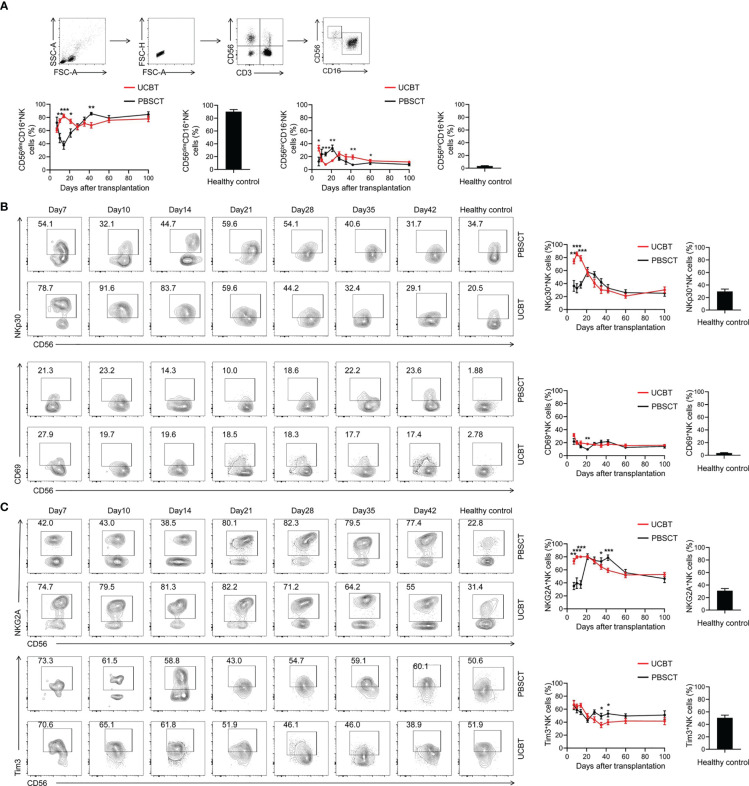
The dynamic changes in the NK cell subpopulations and the expression levels of immune-activating molecules on the NK cells. **(A)** The frequencies of NK cell subpopulations during the early posttransplant period after UCBT and PBSCT. Representative flow cytometry contour plots and the dynamic changes in the expression levels of **(B)** NKp30, CD69, **(C)** NKG2A and Tim3 in the gated NK cells during the early posttransplant period after UCBT and PBSCT. The histogram shows data for the healthy control group. The data was analyzed by the Mann–Whitney U test and represented as means ± SEM. *p < 0.05; **p < 0.01; ***p < 0.001.

NK cells are the first subpopulation of lymphocytes that are reconstituted after HSCT, and play a key role in GVL and other immune responses (13). Therefore, we characterized NK-cell reconstitution after HSCT by analyzing the immature CD56brightCD16− NK cells and mature CD56dimCD16+ NK cells. The results demonstrated that the immature NK cell subsets expanded during the first three weeks after transplantation in the PBSCT group (**Figure 6A**). This result was consistent with previous reports (35). However, the UCBT group showed increased expansion of the mature NK cells after transplantation.

The activity of NK cells is dependent on the balance between the immune-activating and immunosuppressive receptor signals expressed on NK cell (36). Therefore, we analyzed the differences in the expression levels of immune-activating molecules, NKP30 and CD69, and immunosuppressive molecules, NKG2A and Tim-3, on NK cells. The UCBT group showed significantly higher expression of NKP30 during the first two weeks after transplantation compared to the PBSCT group; the expression levels of NKP30 decreased gradually after the first 2 weeks of transplantation (**Figure 6B**). CD69 expression levels were higher in the HSCT patients than in the healthy subjects during the first 100 days, with higher CD69 expression levels in the NK cells of the UCBT group on day 21 after transplantation (**Figure 6B**). The immunosuppressive marker, NKG2A and Tim-3, was overexpressed in the UCBT group during the first 3 weeks after HSCT; however, the expression levels of NKG2A and Tim-3 in the NK cells was significantly lower in the UCBT group compared to the PBSCT group in the fifth and sixth weeks after transplantation (**Figure 6C**).

Collectively, these results showed that the NK cells in the UCBT group were of a higher activation state compared to the PBSCT group. Moreover, the UCBT group showed higher frequency of mature CD56dimCD16+ NK cells.

### The plasma levels of GM-CSF were higher in the patients that underwent UCBT

The above results showed that there were significant differences in the early activation status of the immune cells and the differentiation into various subpopulations between the UCBT and the PBSCT groups of patients, especially in the first three weeks after transplantation.

**Figure 7:**
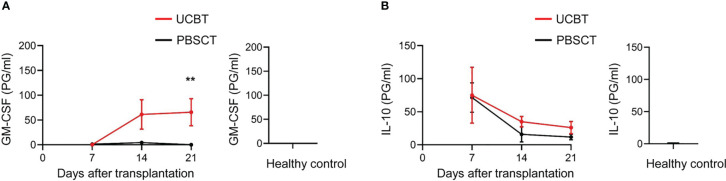
GM-CSF levels are elevated in patients after UCBT. ELISA assay results show the plasma levels of **(A)** GM-CSF and **(B)** IL-10 in the UCBT (n=9) and PBSCT (n=9) group of patients at different time points after HSCT. The histogram shows the data for the healthy control group (n=14). The data was analyzed by the Mann-Whitney U test and represented as means ± SEM. **p < 0.01.

Previous studies have established a significant role for the cytokines such as IL-10 and GM-CSF in the pathogenesis of PES and aGVHD (17–19, 21). Therefore, we investigated the dynamic changes in the plasma levels of IL-10 and GM-CSF on days 7, 14, and 21 after HSCT. We found that the levels of the pro-inflammatory cytokine, GM-CSF, were significantly higher in the UCBT group on day 21 after transplantation compared to the PBSCT group (**Figure 7A**). Furthermore, GM-CSF was not detected in the plasma samples of healthy subjects. However, plasma levels of IL-10 were similar in the UCBT and PBSCT groups (**Figure 7B**).

## Discussion

Allogeneic HSCT is an effective cure to restore the normal levels and functions of hematopoietic and immune cells in patients with disorders of the hematopoietic system or the immune system (37). The degree of immune reconstitution and functional recovery in the HSCT patients determines the manifestation of GVL effects, GVHD, infections, and transplantation-related mortality, all of which subsequently affect the clinical outcomes and quality of life of patients that undergo HSCT (38). Immune reconstitution after HSCT is highly variable among patients. Therefore, in this study, we compared the degree of immune reconstitution between patients that underwent UCBT or PBSCT. Our study demonstrated that patients that underwent UCBT were associated with significantly higher degree of early immune reactions. This was in concordance with previous findings (20). Subsequently, we compared the immune cells characteristics of patients that underwent UCBT and PBSCT.

Firstly, we analyzed the reconstitution of the CD4^+^ and CD8^+^ T cell subpopulations. The differentiation of naïve CD4^+^ T cells into Th1 and Th17 cells is implicated in the pathophysiology, initiation, and aggravation of aGVHD (39). Previous studies have shown that the allogeneic reactive naïve CD4^+^ T cells from the donor are responsible for GVHD by attacking the recipient’s tissue (40). In our study, patients that underwent UCBT showed a higher frequency and number of naïve CD4^+^ T cells. Previous studies have shown that CD4^+^CD25^+^Foxp3^+^ T cells are relatively abundant in the cord blood and significantly inhibit NK cell and T cell functions (41, 42). However, our findings showed that the frequency and numbers of Treg cells after UCBT were significantly lower compared to the patients that underwent PBSCT. Furthermore, our results showed increased expansion of the CD56^dim^CD16^+^ NK cell subpopulation in the UCBT group. These results demonstrated that the higher numbers of naïve CD4^+^ T cells and CD56^dim^CD16^+^ NK cells and lower numbers of Treg cells may play a key role in the higher proportion of early immune reactions observed in patients that underwent UCBT.

Secondly, we analyzed the expression levels of immune-activating and immunosuppressive molecules on the CD8^+^ T cells and NK cells. Our data showed high expression of CD38 and CD69 on the CD8^+^ T cells and NKP30 and CD69 on the NK cells during the initial stages after transplantation. These characteristics were more pronounced in patients that underwent UCBT, thereby suggesting greater activation of the CD8^+^ T cells and the NK cells. Furthermore, the expression levels of granzyme B were significantly higher in the CD8^+^ T cells of patients that underwent UCBT, which suggested increased cytolytic function of the CD8^+^ T cells. In patients that undergo HSCT, high expression levels of immunosuppressive receptors such as PD-1, Tim-3, and NKGA2 are observed on the T cells and the NK cells. Previous studies have shown that the depletion of lymphocytes induces homeostatic proliferation of T cells and the up-regulation of PD-1 and Tim-3 expression on the T cells (43, 44). Therefore, we speculate that the high expression of inhibitory receptors during the initial stages after transplantation prevents hyperactivation of the T cells and NK cells and helps maintain immune system homeostasis. Furthermore, the high expression of both immune-activating and immunosuppressive receptors on the T cells and the NK cells in patients that underwent UCBT suggests stronger plasticity of the T cells and the NK cells in the UCBT group. Finally, we detected higher levels of the pro-inflammatory cytokine GM-CSF in the UCBT group on day 21 after HSCT. However, we did not further investigate the source of GM-CSF.

In summary, patients that underwent UCBT showed stronger GVL and GVHD effects than those that underwent PBSCT using myeloablative conditioning regimen without ATG. Furthermore, the UCBT and the PBSCT group of patients showed significant differences regarding the reconstitution of CD4+, CD8+, and NK cells and their subpopulations. These changes play a significant role in the adverse immune reactions observed during the initial stages after HSCT. However, clarifying the causal relationship between differences in immune reconstitution and PES/ES/GVHD is very difficult. This is because the occurrence of PES/ES affects implantation and significantly increases the occurrence of grade II-IV aGVHD [16,19]. Once PES/ES occurs, hematologists treat promptly with immunosuppression and aggressively prevent the development of grade II-IV aGVHD to improve transplantation outcomes, but this alters the pattern of immune reconstitution. Considering that most immune assessments will be later than drug therapy, more rational research protocols need to be explored to elucidate this dilemma. Overall, our findings might provide new insights into understanding the causal relationship between early immune responses and immune cell reconstitution after HSCT.

## Data availability statement

The raw data supporting the conclusions of this article will be made available by the authors, without undue reservation.

## Ethics statement

The studies involving human participants were reviewed and approved by The Ethics Committee of the University of Science and Technology of China. Written informed consent to participate in this study was provided by the participants’ legal guardian/next of kin.

## Author contributions

XuZ and WW designed and conducted experiments, analyzed data, and wrote the manuscript. DW and SN performed the experiments and interpreted the data. PQ, KS and GS helped to analyze the data. WY, XiZ and LG helped to collected whole-blood samples and patient information. DW and HL designed the study, supervised the research, and revised the manuscript. All authors contributed to the article and approved the submitted version.
