# Supplementary material for: Dynamic comparison of early immune reactions and immune cell reconstitution after umbilical cord blood transplantation and peripheral blood stem cell transplantation
Source: Front Immunol. 2023 Apr 11;14:1084901. doi: 10.3389/fimmu.2023.1084901 (PMC10126295; doi:10.3389/fimmu.2023.1084901)
Supplement: Supplementary file 1 [file DataSheet_1.docx]

**Supplementary table 1. Antibodies used in this study.**

| **Antibody Names** | **Catalog Numbers** | **Source** |
| --- | --- | --- |
| Brilliant Violet 510^TM^ anti-human CD45 | 304036 | Biolegend |
| APC/Cyanine7 anti-human CD3 | 344818 | Biolegend |
| Brilliant Violet 421 ^TM^ anti-human CD56 | 362552 | Biolegend |
| Alexa Fluor 700 anti-human CD16 | 302026 | Biolegend |
| Brilliant Violet 510 ^TM^ anti-human CD8a | 300934 | Biolegend |
| Brilliant Violet 570 ^TM^ anti-human CD4 | 300534 | Biolegend |
| Brilliant Violet 605 ^TM^ anti-human CD45RA | 304134 | Biolegend |
| PerCP/Cyanine5.5 anti-human CD62L | 04824 | Biolegend |
| FITC CD159a(NKNG2A) | 5210901936 | Biolegend |
| P/Cyanine7 anti-human CD337(NKP30) | 325214 | Biolegend |
| PE anti-human CD366(Tim-3) | 345006 | Biolegend |
| FITC anti-human CD4 | 300506 | Biolegend |
| APC anti-human CD25 | 302610 | Biolegend |
| PE anti-human FOXP3 | 320108 | Biolegend |
| BV421 anti-human Granzyme B | 563389 | BD Biosciences |
| PE/Cyanine7 anti-human CD8 | 980910 | Biolegend |
| Brilliant Violet 421^TM^ anti-human CD279（PD-1） | 367422 | Biolegend |
| PerCP/Cyanine5.5 anti-human CD38 | 303522 | Biolegend |
