# Supplementary material for: Dynamic comparison of early immune reactions and immune cell reconstitution after umbilical cord blood transplantation and peripheral blood stem cell transplantation
Source: Front Immunol. 2023 Apr 11;14:1084901. doi: 10.3389/fimmu.2023.1084901 (PMC10126295; doi:10.3389/fimmu.2023.1084901)
Supplement: Supplementary file 2 [file DataSheet_2.docx]

**Dynamic comparison of early immune reactions and immune cell reconstitution after umbilical cord blood transplantation and peripheral blood stem cell transplantation**

Xuxu Zhao^1,3†^, Wenya Wang^1,3†^, Shiqin Nie^1,3^, Liangquan Geng^1^, Kaidi Song^1^, Xinyi Zhang^1^, Wen Yao^1^, Ping Qiang^1^, Guangyu Sun^1^, Dongyao Wang^1,3,4†^*, Huilan Liu^1,2,3^*

1 Department of Hematology, the First Affiliated Hospital of USTC, Division of Life Sciences and Medicine, University of Science and Technology of China, Hefei, Anhui, 230001, China.

2 Department of Transfusion, the First Affiliated Hospital of USTC, Division of Life Sciences and Medicine, University of Science and Technology of China, Hefei, Anhui, 230001, China.

3 Blood and Cell Therapy Institute, Division of Life Sciences and Medicine, University of Science and Technology of China, Hefei, Anhui, 230001, China.

4 Anhui Province Key Laboratory of Immunology in Chronic Diseases, Bengbu Medical College, Bengbu, Anhui 233030, China.

† These authors have contributed equally to this work.

***Correspondence**: Huilan Liu, (E-mail: huilanl@ustc.edu.cn), Dongyao Wang, (E-mail: [dywsn@ustc.edu.cn](mailto:dywsn@ustc.edu.cn)). Division of Life Sciences and Medicine, University of Science and Technology of China, 443 Huangshan Road, Hefei city 230027, Anhui, China; Tel: 86-551-6228-3347; Fax: 86-551-6228-3730.

**Supplementary Figure and Figure Legend**


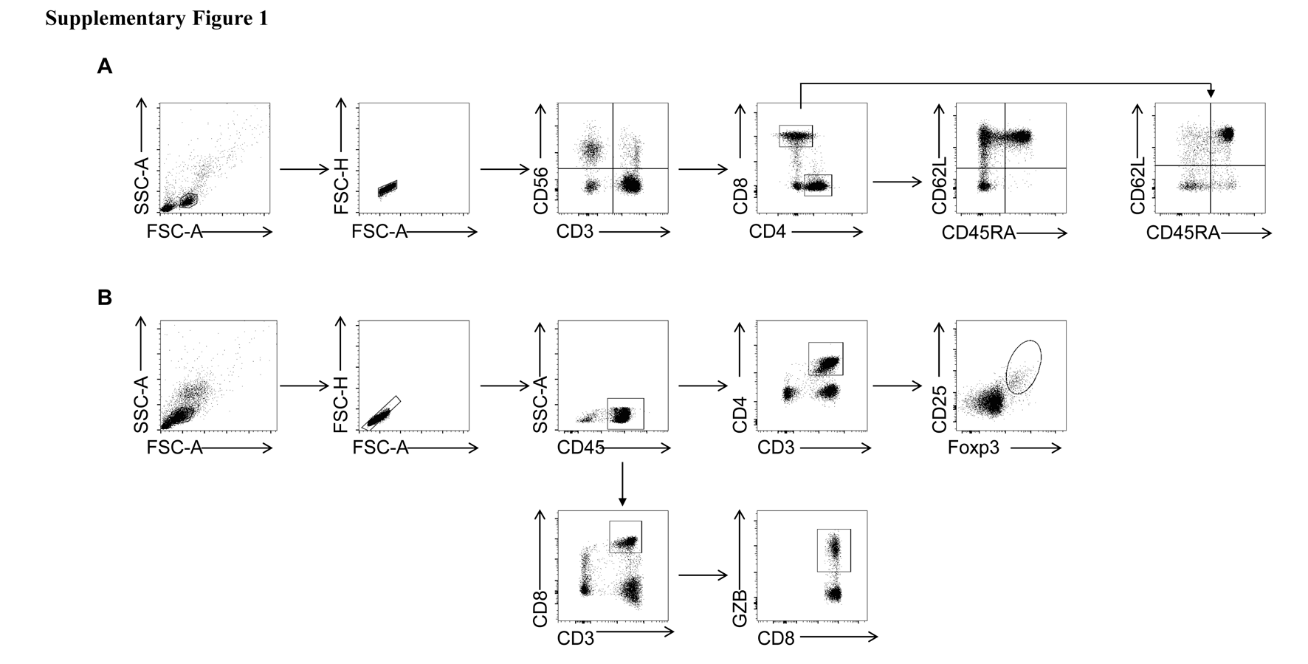


**Supplementary Figure 1.** **Flow cytometry gating strategy.**

**(A)** Gating strategy for the CD4^+^ T cells and the CD8^+^ T cells subpopulations in the peripheral blood mononuclear cell (PBMC) samples. **(B)** Gating strategy for the Tregs in the PBMC samples.
